# Lessons in immune adaptations to hypoxia revealed by comparative and evolutionary physiology

**DOI:** 10.1186/s12915-023-01788-8

**Published:** 2023-12-29

**Authors:** Erica C. Heinrich, Michael S. Tift

**Affiliations:** 1 Division of Biomedical Sciences, School of Medicine, University of California Riverside, Riverside, CA USA; 2 University of North Carolina Wilmington, 601 S. College Rd., Wilmington, NC 28403 USA

## Abstract

**Recent findings from comparative and evolutionary physiologists reveal novel insights into the regulation of inflammation and immune function under conditions of chronic-sustained and intermittent hypoxia. Comparative approaches provide a valuable gateway for discovering essential principals of physiology and adaptive molecular strategies utilized in nature that protect against clinically relevant stressors, which can guide therapeutic developments in biomedical science.**

## Clinical importance of hypoxia-induced inflammation and immune modulation

Hypoxia is a critical element of several chronic and acute illnesses and injuries. Hypoxemia and downstream tissue hypoxia due to hypoperfusion, anemia, or diffusion limitation in acute and chronic lung disease not only have direct impacts but also modulate systemic immune function and local tissue inflammatory signaling. The influence of hypoxia on cellular function has been of particular interest in the field of cancer biology, leading to major advancements in the understanding of tumor pathology and therapeutic approaches. However, outside of this field, it is uncommon that studies of clinical immune pathologies include an evaluation of the independent and interactive impact of hypoxia, even though hypoxia, inflammation, and immune activation typically occur in tandem in vivo. Indeed, initial inflammatory processes during infection and injury contribute to, and occur in response to, disrupted vasculature, immune cell recruitment, fluid retention, and other factors that limit the rate of oxygen delivery to tissue.

## Lessons from comparative and evolutionary physiology

Throughout human evolutionary history, most occurrences of tissue hypoxia likely resulted from consequences of injury and infection, which may have driven adaptations that linked hypoxia signaling to protective immune responses and tissue repair. As a result, the molecular mechanisms that control the transcriptional responses to hypoxia and inflammatory stimuli are tightly linked. These mechanisms also contribute to maladaptive pathologies in the modern era in which hypoxia occurs in the absence of infection or acute tissue injury. For example, tissues may experience pathologic hypoxia in the context of chronic lung disease or in response to tissue hypoxia that is secondary to impaired tissue perfusion in chronic inflammatory and metabolic disorders such as diabetes and inflammatory bowel disease.

In contrast to humans, many animal species encounter severe and prolonged bouts of hypoxemia as a function of their normal behavior. Marine mammals routinely dive for durations that range from several minutes to hours, during which heart rates can drop to as low as a few beats per minute, peripheral tissues become ischemic, and arterial oxygen partial pressures can decline to levels seen in humans breathing ambient air on the summit of Mt. Everest and below those known to cause shallow water blackout in humans [1]. Once they return to the surface and begin breathing again, heart rate increases, peripheral tissues are reperfused, and arterial oxygen partial pressures are restored. As a result, these animals developed distinct physiological adaptations to protect tissues against chronic intermittent hypoxia. Many of these adaptations extend to protect against hypoxia-induced inflammation and oxidative stress. For example, the serum from Weddell and elephant seals has distinct anti-inflammatory properties that may help protect tissues against diving induced hypoxic injuries [2]. Certain deep-diving mammals have also developed strategies to cope with increased oxidative stress associated with repeated ischemia–reperfusion events from diving, including elevated levels of endogenous antioxidants and carbon monoxide, mitochondrial adaptations, and dampened angiogenic signaling [3, 4].

Burrowing mammals also routinely experience conditions of hypoxia and hypercapnia, as there is typically a reduction in fresh air that can penetrate the burrows to replenish oxygen consumed by the animals and remove carbon dioxide. Because of this, these species have also developed traits which promote hypoxia and hypercapnia tolerance. Naked mole rats are known to be one of the most hypoxia tolerant mammalian species, and perhaps as a function of their hypoxia-tolerant traits, they are known to be cancer resistant, have prolonged lifespans, and demonstrate unique immune phenotypes. For example, these animals exhibit a novel neutrophil subset that expresses several antimicrobial factors at very high levels. Additionally, single-cell sequencing revealed a high myeloid-to-lymphoid cell ratio and a lack of canonical natural killer cells [5]. These findings challenged the traditional understanding of mammalian immunity and suggested that naked mole rats favor a myeloid-based mode of innate immunosurveillance.

Many wild species also evolved to thrive in high-altitude regions where low atmospheric pressure results in exposure to a chronically hypoxic environment. Much work over the past decade has illuminated adaptations within high-altitude native species, including modifications in hemoglobin-oxygen binding affinity and its interactions with allosteric effectors across many species of birds and mammals [6]. Similarly, adaptive changes in lung function and the neural control of breathing were identified in high-altitude rodents [7]. The genetic underpinnings of these adaptations have been examined as well, highlighting the importance of hypoxia-inducible factor gene variants in the survival of high-altitude species. Carefully designed cross-over experiments in high-altitude native animals that are reared under controlled laboratory conditions can be used in combination with population genomic surveys to clearly reveal the independent roles of genetic and environmental drivers on physiological responses to hypoxia.

These are only a few examples of unique adaptations to hypoxia across wild vertebrate species which lead us toward understanding the unifying principles of adaptive responses to hypoxic stress. These hypoxia-tolerant phenotypes developed naturally over millions of years of evolution using the same molecular building blocks available as therapeutic targets in humans. As a result, future research should further investigate the molecular underpinnings of these adaptations so that these strategies can perhaps be adopted as targets for therapeutic interventions.

## Revitalizing comparative approaches to biomedical science

Biomedical research aims to identify mechanisms underlying clinical pathologies, with the goal of developing novel treatments. In most cases, these mechanisms are examined in commercially available rodent models due to ease of use, homogeneous genetic backgrounds, and a strong foundation of previous research in these animals. While it is undeniable that these approaches are valuable, we can learn a great deal by examining the unique and elegant mechanisms that evolved in wild animal models that have responded and adapted to clinically relevant environmental and ecological stressors like hypoxia. Identifying these latent mechanisms can stimulate creative ideas for innovative therapeutic approaches that cannot be identified in traditional rodent models.

This approach embodies the spirit of the Krogh Principal of comparative physiology. In 1929, August Krogh wrote “For a large number of problems there will be some animal of choice or a few such animals on which it can be most conveniently studied.” Krogh believed that if it were possible to obtain an understanding of “essential characteristics of matter in the living state” that we must study vital physiological functions “in all their aspects throughout the myriads of organisms” [8]. Krogh understood that to successfully comprehend the mechanisms behind universal physiological concepts tied to human health and medicine, there would need to be decades of research with unique model systems to properly study certain questions. Consequently, this philosophy guided the larger field of physiology until recent decades.

Indeed, many of the most important discoveries in physiology and medicine were revealed through studies of diverse and unique animal models. For example, by examining pH-temperature relationships across a diversity of organisms, including ectotherms, with body temperatures spanning a much larger range than mammals alone, the “alphastat hypothesis” was revealed. This was a groundbreaking hypothesis which helped to eventually explain why protein function was conserved in conditions of changing temperature and how proteins respond to changes in isothermal pH. In fact, the observations in ectotherms corrected an erroneous view that many proteins functioned only within a narrow range of pH around 7.4. Another example of contributions of comparative physiology to medicine came from Krogh himself, who received a Nobel Prize for elucidating the structure and function of capillaries based on studies in another ectotherm, frogs. Krogh examined capillary networks in the frog tongue because of its translucent color (which allowed direct view of the capillaries and their associated arteries and veins) as well as the highly developed capillary tone in this tissue.

Unfortunately, in recent years, the fields of biomedical science and comparative physiology seem to have lost their intimate link. However, as we reach the centennial of Krogh’s evaluation of “The Progress of Physiology,” perhaps it is time to revisit more diverse experimental approaches. It has become clear that many discoveries made in rodent models are not replicated in humans, leading to low rates of successful translation to clinical trials [9]. In fact, many studies in rodent models are not successfully replicated by separate research teams using the same models and experimental design, due to lack of rigor in study design and insufficient considerations of statistical power. This is not a new problem, as Krogh himself proposed in 1929 that the field of physiology was beginning to suffer from a lack of critical thought in experimental design and hypothesis formulation, which lead to early signs of an unwieldy literature, poor replication, and waste of resources and animals. One solution to the overuse of animals in research is to choose better models geared toward answering a particular question. These models may include in vitro human cell-based assays or novel animal models which either better represent the human phenotype or provide unique insight into a question because of specific adaptations in that model.

Many comparative physiologists have begun reemphasizing the importance of approaching biomedical questions by examining physiological solutions to challenging environments that already exist in nature [10]. This philosophy extends not only to diverse animal models but also within-group comparisons by examining physiological plasticity and long-term adaptation to various stressors. Indeed, studies with humans exposed to extreme environments, as well as human populations that have undergone evolutionary adaptation to these environments, such as high-altitude native groups on the Tibetan Plateau, Andean Altiplano, and Ethiopian highlands, can be informative for discovering protective mechanisms against hypoxia that already exist and can provide insight into novel therapeutic targets.

In conclusion, many lessons can be learned about human pathologies and treatment options by studying wild animal models. Many species, some mentioned above, are known to avoid certain injuries or diseases that humans or other species would be susceptible to. These species provide a unique opportunity to advance our understanding of both human and veterinary medicine. With the advancement of technology, capacity to study these species in controlled settings, and ability to link genotypes and phenotypes, new techniques can be used to address previously unanswered questions. Biomedical research should embrace a comparative approach to identify these unique mechanisms and test their therapeutic potential.

## Data Availability

Not applicable.
